# Immune Profiling Panel: A Proof-of-Concept Study of a New Multiplex Molecular Tool to Assess the Immune Status of Critically Ill Patients

**DOI:** 10.1093/infdis/jiaa248

**Published:** 2020-07-21

**Authors:** Dina M Tawfik, Laurence Vachot, Adeline Bocquet, Fabienne Venet, Thomas Rimmelé, Guillaume Monneret, Sophie Blein, Jesse L Montgomery, Andrew C Hemmert, Alexandre Pachot, Virginie Moucadel, Javier Yugueros-Marcos, Karen Brengel-Pesce, François Mallet, Julien Textoris

**Affiliations:** 1 EA7426 “Pathophysiology of Injury-Induced Immunosuppression,” PI3, Université Claude Bernard Lyon-1 Hospices Civils de Lyon, bioMérieux, Lyon, France; 2 Open Innovation and Partnerships, bioMérieux, Lyon, France; 3 Immunology Laboratory, Hospices Civils de Lyon, Edouard Herriot Hospital, Lyon, France; 4 Anaesthesia and Critical Care Medicine Department, Hospices Civils de Lyon, Edouard Herriot Hospital, Lyon, France; 5 BioFire Diagnostics LLC, Salt Lake City, Utah, USA

**Keywords:** critically ill patients, sepsis, multiplex PCR, biomarkers, in vitro diagnostic, FilmArray, immune response, syndromic panel

## Abstract

**Background:**

Critical illness such as sepsis is a life-threatening syndrome defined as a dysregulated host response to infection and is characterized by patients exhibiting impaired immune response. In the field of diagnosis, a gap still remains in identifying the immune profile of critically ill patients in the intensive care unit (ICU).

**Methods:**

A new multiplex immune profiling panel (IPP) prototype was assessed for its ability to semiquantify messenger RNA immune-related markers directly from blood, using the FilmArray System, in less than an hour. Samples from 30 healthy volunteers were used for the technical assessment of the IPP tool. Then the tool was clinically assessed using samples from 10 healthy volunteers and 20 septic shock patients stratified using human leukocyte antigen–DR expression on monocytes (mHLA-DR).

**Results:**

The IPP prototype consists of 16 biomarkers that target the immune response. The majority of the assays had a linear expression with different RNA inputs and a coefficient of determination (*R*^2^) > 0.8. Results from the IPP pouch were comparable to standard quantitative polymerase chain reaction and the assays were within the limits of agreement in Bland–Altman analysis. Quantification cycle values of the target genes were normalized against reference genes and confirmed to account for the different cell count and technical variability. The clinical assessment of the IPP markers demonstrated various gene modulations that could distinctly differentiate 3 profiles: healthy volunteers, intermediate mHLA-DR septic shock patients, and low mHLA-DR septic shock patients.

**Conclusions:**

The use of IPP showed great potential for the development of a fully automated, rapid, and easy-to-use immune profiling tool. The IPP tool may be used in the future to stratify critically ill patients in the ICU according to their immune status. Such stratification will enable personalized management of patients and guide treatments to avoid secondary infections and lower mortality.

Critically ill patients in the intensive care unit (ICU) exhibit a high risk of morbidity and mortality and require special care and timely interventions. One of the major life-threatening situations in the ICU is sepsis, which is defined as an organ dysfunction caused by a dysregulated host response to infection [1]. This dysregulated response includes an unbalanced pro- and anti-inflammatory immune response that translates into various immune profiles. These profiles manifest as a state of hyperinflammation or profound immunosuppression [2].

The current understanding of the underlying pathophysiology of sepsis has encouraged clinicians to use targeted therapy in clinical trials to potentially restore the immune homeostasis and prevent unfavorable outcomes [3, 4]. For more than 20 years, researchers have described several biomarkers in different platforms to characterize the immune dysfunctions of sepsis [5–8]. Several studies found that low expression of human leukocyte antigen–DR on monocytes (mHLA-DR) was repeatedly associated with increased risk of acquiring secondary infections and mortality [9, 10]. Low expression of mHLA-DR can be used as a surrogate marker for monocyte anergy and decreased antigen presentation, which might contribute to sepsis-induced immunosuppression [11]. The information provided by biomarkers such as HLA-DR and transcriptomic biomarkers could be used to determine the immune status of a patient and become a potential target for novel immunomodulatory therapies. For instance, immune-stimulatory agents such as interleukin (IL) 7 [12], granulocyte macrophage-colony stimulating factor (GM-CSF) [13], and interferon gamma (IFN-γ) [14–16] could restore lymphocyte and monocyte function and reverse sepsis-induced immunosuppression. Other immunomodulatory therapies could aim to dampen the cytokine storm such as IL-1 receptor antagonist (IL-1Ra) [17] and anti–IL-6 [18]. Nonetheless, personalized care is impeded by the absence of a comprehensive and fast diagnostic tool that would allow clinicians to precisely monitor patients’ immune status.

The immune pathogenesis in sepsis is complex and heterogeneous, and more than 1 biomarker is needed to address the different facets of the immune system that could risk-stratify patients and guide therapy [19, 20]. Transcriptomic gene signatures were identified and are good candidates to stratify septic patients according to severity and worsening of outcomes, which could be used to guide therapy [21–23]. Research studies endorsed that patient stratification can be achieved by the use of gene expression profiling in a rapid multiplex diagnostic tool [24]. However, all of the current immune profiling attempts are still in their infancy due to the complexity of the available platforms [19]. The recent advances in multiplex polymerase chain reaction (PCR) technology could enable the deployment of panels of biomarkers for diagnosing and stratifying patients at the bedside [25, 26]. Multiplex molecular platforms such as the FilmArray System (BioFire Diagnostics, LLC) have been developed and several commercial kits are available on the market, enabling the accurate detection of pathogens in less than an hour [27]. FilmArray is certified by the Food and Drug Administration, United States and the In-Vitro Diagnostic Medical Device Directive, Europe. FilmArray is a fully automated, user-friendly multiplex-nested qPCR (quantitative Polymerase Chain Reaction) technology that can measure up to 45 assays with a simplified report as a readout [28]. We present here a proof-of-concept study for an immune profiling panel (IPP), a transcriptomic molecular tool assessing the immune status directly from whole blood. A panel of markers were selected to target immune functions such as monocyte anergy, antigen presentation, lymphocyte exhaustion, cytokines, and regulatory pathways. We report the technical studies of the first IPP prototype used for the semiquantification of messenger RNA (mRNA) from whole blood in the FilmArray System. The panel was tested on samples from septic shock patients stratified according to the expression of mHLA-DR, as a decrease in the expression of mHLA-DR has been linked to poor outcomes [29].

## MATERIALS AND METHODS

### Immune Profiling Panel

**Figure 1.**
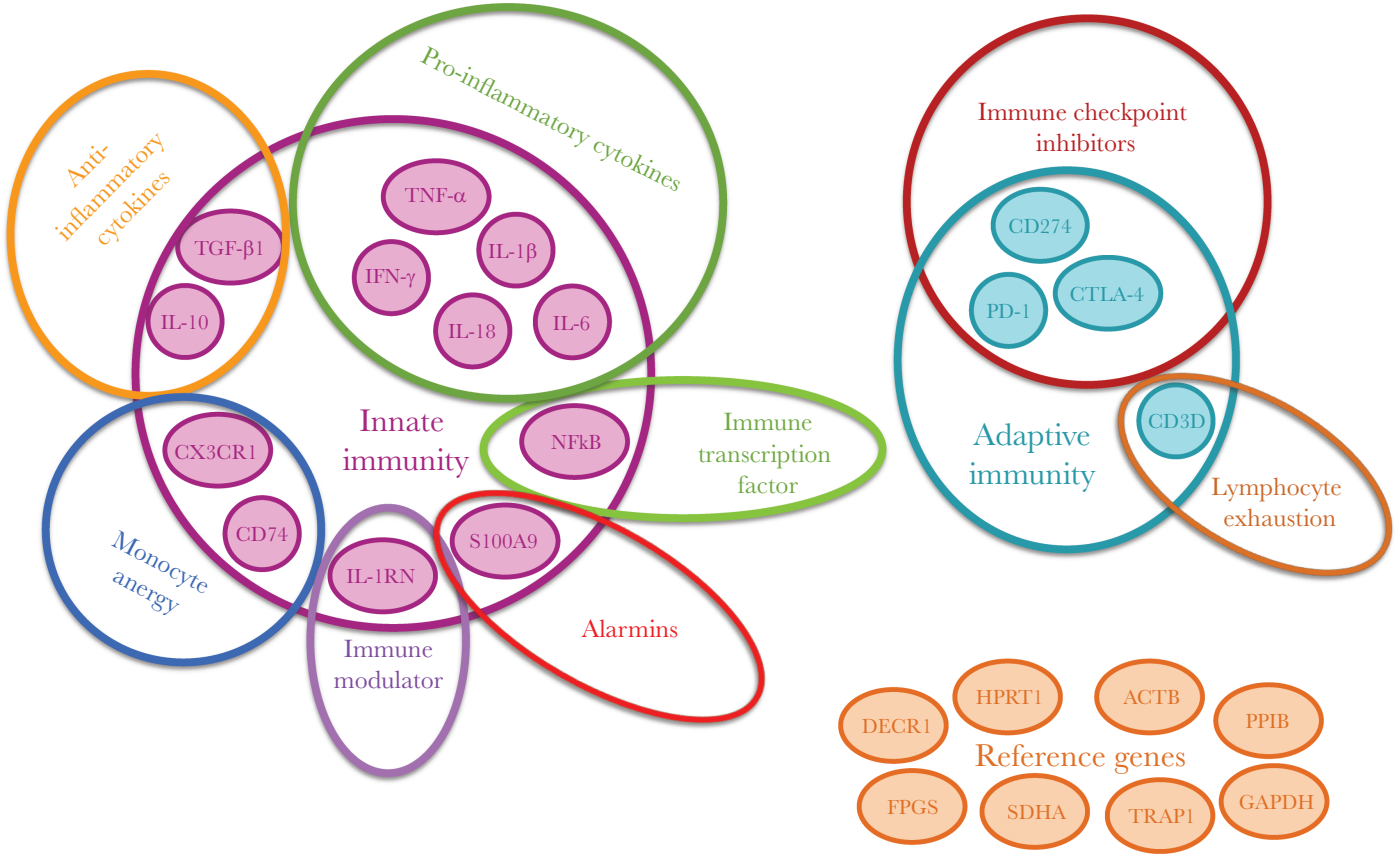
The immune profiling panel. The figure illustrates the selected markers of the panel that includes 16 target genes and describes the different pathways targeted. The panel also features 8 reference genes for signal normalization. The panel of markers was selected to target different arms of the immune responses (innate and adaptive), several immune functions (pro- and anti-inflammatory cytokines) and immune pathways.

Selection of IPP markers was based on (1) previous laboratory expertise in evaluating the performance and robustness of immune-related markers in clinical trials [22–24]; (2) relevant data on prognostic immune-related markers in literature [7, 25]; (3) the technical performance of the selected assays in duplex and multiplex qPCR; and (4) addressing a balanced representation of the pathways involved in various cells of both arms of the immunity. Overall, IPP covers genes coding for soluble markers of the inflammatory response (pro- and anti-inflammatory), transcription factors, and membrane markers associated to the innate and adaptive responses, as well as alarmins and apoptosis markers (Figure 1). The first prototype of IPP pouches encompasses 16 target assays and 8 reference genes, for signal normalization. Several lots of IPP pouch prototypes were manufactured by BioFire Diagnostics and transferred to our facility for technical assessment. The supplied IPP pouches contain all the biochemical reagents and primers lyophilized and ready to use upon hydration, which is done by injecting 1 mL of hydration solution provided with the kit. One hundred microliters of whole blood sampled on PAXgene tube was mixed with approximately 800 µL of the lysis buffer provided with the panel and directly injected into the pouch, where a volume of 300 µL of the mix is automatically drawn into the first well [30]. Then the pouches were inserted into the FilmArray 2.0 instrument (BioFire) where nucleic acids are automatically extracted from the sample, then RNA is reversed transcribed and amplified in first-stage multiplex PCR followed by second-stage quantitative nested PCR (Supplementary Figure 1) [28]. In some experiments, extracted RNA samples were tested directly in the pouches. A controlled uniform RNA input helped us to evaluate the semiquantitative ability of the platform and assess the success of the signal normalization. Results from IPP prototype pouches are delivered in less than 1 hour in the form of real-time quantification cycle (Cq) values and postamplification melt peaks. This is different from the commercial kits that provide an easy-to-read report generated by an internal interpretation algorithm, not yet available for the IPP prototype.

### Healthy Volunteers and Patient Samples

#### Healthy Volunteers

Whole blood from healthy volunteers collected in PAXgene tubes (Pre-Analytix, Germany) was obtained from the Etablissement Français du Sang (EFS; French blood bank, Grenoble). PAXgene tubes were inverted several times and incubated for 2 hours at room temperature according to the manufacturer’s recommendation.

Total RNA was manually extracted from 30 healthy volunteers using PAXgene blood RNA kit (Pre-Analytix) according to the manufacturer’s instructions. The extracted RNA’s quantity and quality were determined using a Nanodrop ND-1000 spectrophotometer (Nanodrop Technologies) and an Agilent 2100 bioanalyzer (Agilent Technologies) to compute the RNA integrity number. For the linearity study, 10 extracted RNA samples with different inputs (0.5, 1, 2, 10, and 100 ng) were directly injected in the amplification chamber of the IPP pouch and samples were run on FilmArray to study the linearity of the nested PCR assays. RNA of 10 healthy volunteers was tested against septic shock patients’ samples at a quantity input of 10 ng.

#### Patients With Septic Shock 

RNA samples from patients were obtained from a previous prospective study, ImmunoSepsis-1, which included adults with septic shock enrolled from December 2001 to April 2005 from 2 French university hospital ICUs [31, 32]. Twenty RNA samples collected from patients on day 3 were selected according to the expression of mHLA-DR measured by flow cytometry (Table 1). Ten septic shock patients with an mHLA-DR expression < 30% were selected as the “low mHLA-DR” group. The other 10 patients exhibited > 30% mHLA-DR expression and were grouped as “intermediate mHLA-DR.”

**Table 1.** Clinical Characteristics of Patients With Septic Shock From the ImmunoSepsis-1 Cohort

Characteristic	Borderline mHLA-DR (> 30%) Patients (n = 10)	Low mHLA-DR (< 30%) Patients (n = 10)
Characteristics of patients		
Age, y, median (IQR)	68 (62–77)	67 (63–75)
Male sex	5 (50)	5 (50)
Comorbidities^a^ (≥ 1)	2 (20)	5 (50)
SOFA score^b^ (day 1), median (IQR)	9 (8–12)	11 (10–12)
SOFA score^b^ (day 3), median (IQR)	10 (8–11)	12 (9–14)
SAPS II^b^ (day 1), median (IQR)	47 (44–54)	60 (51–73)
HLA-DR, % expression on monocytes (days 3–4), median (IQR)	56 (50–62)	15 (12–16)
Type of admission		
Medical	7 (70)	5 (50)
Elective surgery	…	1 (10)
Emergency surgery	3 (30)	4 (40)
Primary site of infection		
Abdominal	4 (40)	4 (40)
Pulmonary	4 (40)	5 (50)
Other	2 (20)	1 (10)
Outcomes		
ICU length of stay, d, median (IQR)	21 (14–31)	22 (12–31)
Survivors at day 28	7 (70)	4 (40)

Data are presented as No. (%) unless otherwise indicated.

*Abbreviations: HLA-DR, human leukocyte antigen–DR; ICU, intensive care unit; IQR, interquartile range; mHLA-DR, human leukocyte antigen–DR in monocytes;* SAPS II, Simplified Acute Physiology Score II; *SOFA,* Sequential Organ Failure Assessment.

^a^Comorbidities include cardiac, hepatic, respiratory, or/and renal comorbidities.

^*b*^ *SAPS II and SOFA scores were measured after 24 hours of ICU stay (day 1 and day 3).*

### Reverse Transcription and Real-Time PCR Amplification

The qPCR was performed in a microplate, where RNA was reverse transcribed to complementary DNA (cDNA) using a SuperScript VILO cDNA Synthesis Kit (Life Technologies) and was ready to be amplified. Bench qPCR reactions were performed for S100A9 on a LightCycler 480 instrument (Roche), using its corresponding probes master kit (Roche) following the manufacturer’s instructions. In brief, PCR reaction was carried out in triplicates in a final volume of 20 μL containing 0.5 μM of primers and 0.1 μM of probe, with an initial denaturation step of 10 minutes at 95°C, followed by 45 cycles of touchdown PCR protocol (10-second denaturation at 95°C, 29-second annealing at 58°C–68°C, and 1-second extension at 72°C). The LightCycler software was used to automatically determine the Cq value for each assay. A prototype Argene kit (bioMérieux) was used for the amplification of CX3CR1. The kits and reverse-transcription PCR (RT-PCR) amplifications were performed with an ABI7500 thermocycler (Applied BioSystems). In brief, sample triplicates were diluted 1:10 and mixed with 15 µL of primer and probe mix, and 0.15 µL of RT was diluted 1:10 in water to a final volume of 25 µL. The PCR protocol included a 5-minute RT step at 50°C for 1 cycle, and Taq polymerase activation step for 15 minutes at 95°C for another cycle. This was followed by PCR protocol of 45 cycles (10-second denaturation at 95°C, 40-second annealing at 60°C, and 25-second elongation step at 72°C). Positive samples presented a signal at 530 nm (FAM); otherwise, the sample was considered negative. Raw Cq values of qPCR and IPP were evaluated for equivalence using Bland–Altman analysis.

### Statistical Analyses

The linearity of markers was evaluated by performing linear regression models of Cq values against the log_10_ transformation of RNA quantities, and the *R*^2^ were reported. Normalized expression values of the genes were expressed as median and interquartile ranges in box-and-whisker plots. Paired Wilcoxon signed-rank test was used to assess significance before and after normalization of the Cq values in 2 RNA quantities. The differential expression of markers between the tested groups was compared using Mann–Whitney *U* test. The level of significance was set at 5% 2-sided tests. Statistical analyses were performed and computed using R software version 3.5.1.

### Ethical Considerations

#### Healthy Volunteers

Informed consent from the blood donors was obtained, and their personal data were anonymized at time of blood donation and before the blood transfer to our facility according to EFS standard regulations for blood donation.

#### Patients With Septic Shock 

The ImmunoSepsis-1 cohort was approved by the local ethics committee (Comité de Protection des Personnes Sud-Est II, institutional review board number 11236). A nonopposition to cohort inclusion was recorded from every patient or the patient’s relative. Since it was a noninterventional trial and complementary blood samples were obtained during patients’ routine blood sampling and tested after the completion of routine follow-up tests, no informed consent was required. The ImmunoSepsis-1 study is registered at the French Ministry of Research and Teaching (number DC-2008–509), at the Commission Nationale de l’Informatique et des Libertés, and on ClinicalTrials.gov (NCT02803346).

## RESULTS

### Repeatability Study

**Figure 2.**
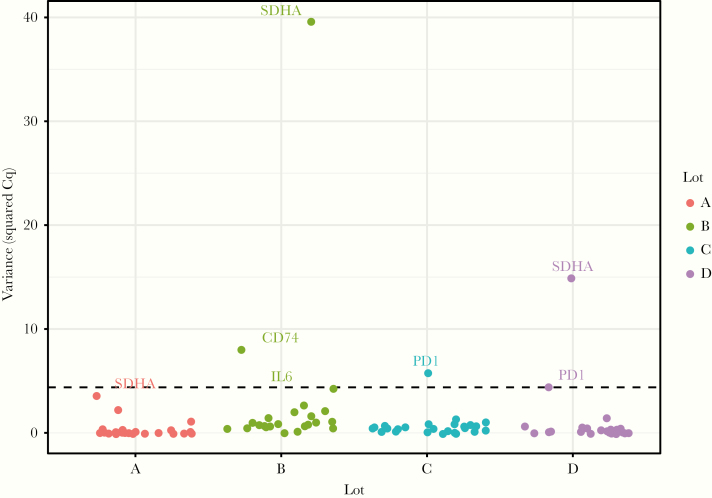
Variability of immune profiling panel (IPP) assays. PAXgene stabilized whole blood from a single healthy donor was tested in triplicates to evaluate the variability of the assay in the IPP tool. The variance in quantification cycle (Cq) values of 4 different lots given as A, B C, and D are presented on the y-axis calculated from the triplicates of the markers’ expression in each lot. Dashed line: cutoff + 1 standard deviation. The names of the target genes above or on the line of variance cutoff are indicated in the plot.

We inspected the repeatability among 4 manufactured prototype lots by testing blood collected from a single healthy donor tested in triplicates. Variance was computed for all the samples and assays in each lot. A threshold of variance acceptance was set to +1 standard deviation (SD) of the overall variance. This threshold was selected as it was more stringent compared to the usually recommended +2 SD and could identify markers with high variance. Figure 2 illustrates that the overall variance for all markers was low across lots. Lot B had the highest variance, which was mainly due to 1 gene (SDHA, a reference gene) that seemed to be also variable in lot D. CD74 was identified as an outlier only in lot B, whereas PD-1 was an outlier in lots C and D. The observed high variances seemed to be assay-related rather than lot-related. In the case of SDHA, the variance might be linked to a problem in primer design that is also observed in the following experiments, while PD-1 variability might be due to its low expression in healthy volunteers. The rest of the assays had minimal variability and remained below the limit of +1 SD, demonstrating the repeatability and robustness of the system.

### Linear Study of IPP Assays

**Figure 3.**
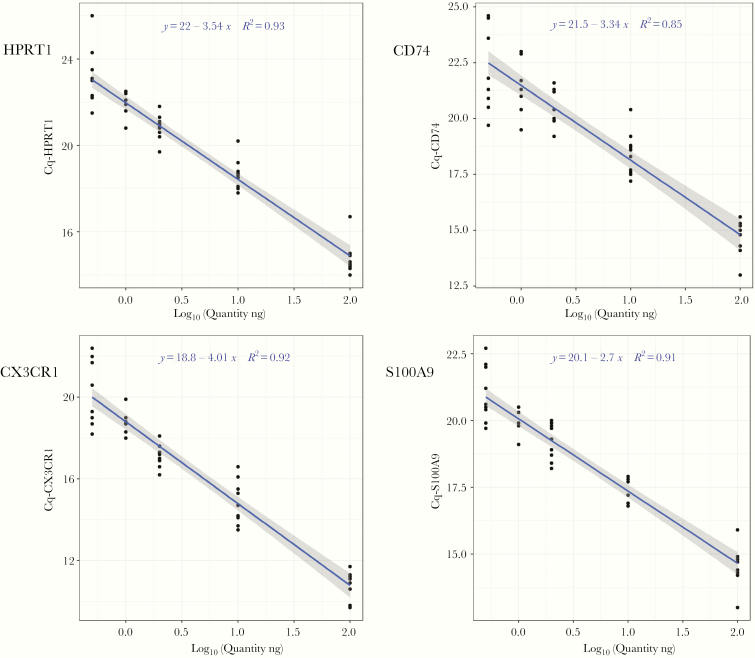
Linearity study of the reference and target assays. Extracted RNA from 10 healthy volunteers was tested in the immune profiling panel using 5 different RNA quantities (0.5, 1, 2, 10, and 100 ng). The linear model of the raw quantification cycle (Cq) values is plotted against log_10_ of the RNA quantities. The slope-intercept equation of each model appears on the plot along with the *R*^2^ values.

The FilmArray platform was initially developed for microbiology applications and the detection of various pathogens from different sample types. Montgomery et al initiated a study to use host response-based assays in FilmArray to discriminate viral from a bacterial infection in patients [33]. In our prototype pouch, we wanted to semiquantify the host immune biomarkers in critically ill patients. To this end, we studied the linearity of the selected assays to ensure the possibility of semiquantification using the FilmArray system. We used 5 known RNA quantities (ranging from 0.5 to 100 ng) to show that IPP markers expression fall into the tested linear range of measurement. Figure 3 illustrates the linearity of 1 reference gene (HPRT1) and 3 target genes (S100A9, CD74, and CX3CR1) representative of the panel. Reference and target assays were found to be linear within the tested range of RNA quantities with *R*^2^ values ranging from 0.51 to 0.94 (median, 0.89). The majority of IPP assays exhibited high *R*^2^ values > 0.8 (Supplementary Table 1). *R*^2^ values < 0.8 were further inspected, such as IL-6 (0.51) and PD-1 (0.63), and may be related to their weak expression in healthy volunteers. Genes such as GAPDH, SDHA, and ACTB showed poor linearity and were discarded from the rest of the analytical studies. Overall, this confirms that IPP assays were linear, which enables the semiquantification of gene.

### Equivalence to qPCR

**Figure 4.**
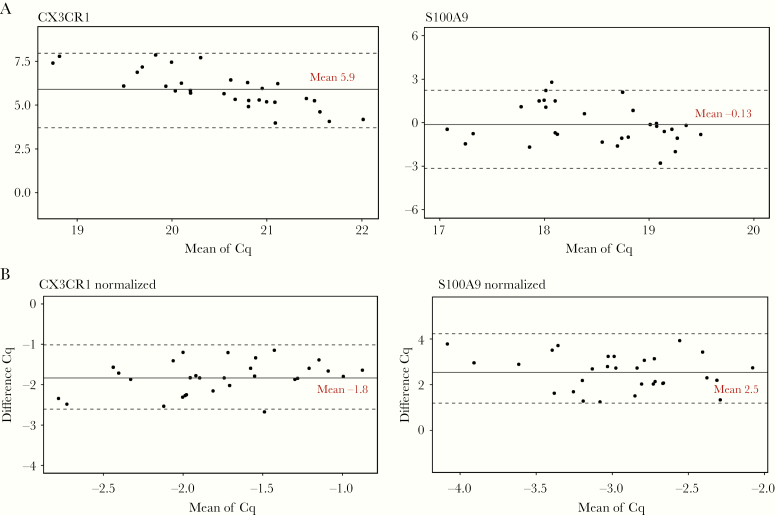
Bland–Altman analysis of 2 target markers expression in the immune profiling panel pouch compared to quantitative polymerase chain reaction (qPCR) results. Whole blood from PAXgene tubes of 30 healthy volunteers’ samples was tested in FilmArray and extracted RNA samples from the same volunteers were tested in qPCR for the equivalence study. The solid horizontal line represents the mean difference and estimates of the systemic bias between the methods; while the dashed lines represents the ± 1.96 SD limits of agreement between the two methods. Both genes are within the limits of agreement, *A*, Raw data. *B*, Normalized. Abbreviation: Cq, quantification cycle.

Several target genes were tested for equivalence between the 2 methods using Bland–Altman analysis. It was observed that 2 assays (CX3CR1 and S100A9) are within the limits of agreement demonstrated as ±1.96 SD calculated from the mean of difference (middle horizontal line) horizontal line. S100A9 was equivalent in both platforms as most of the points are around the mean difference line on the y-axis (Figure 4A), which is close to zero. CX3CR1 presented a higher systematic bias of 5.9 Cq in qPCR compared to IPP and a slight decreasing proportional bias associated with higher Cq values. However, values remained within the limits of agreement (Figure 4A). After normalization of both data and recomputing the Bland–Altman plots, it can be observed that normalization helped eliminate the proportional bias with a slight presence of a systemic bias between the 2 methods for the 2 markers (Figure 4B). Both raw and normalized Cq analyses show that the 2 methods are within the limits of agreement. This analysis demonstrates the concordance between IPP and bench PCR, which is a common reference method used for mRNA quantitation.

### Evaluation of IPP Signal Normalization

**Figure 5.**
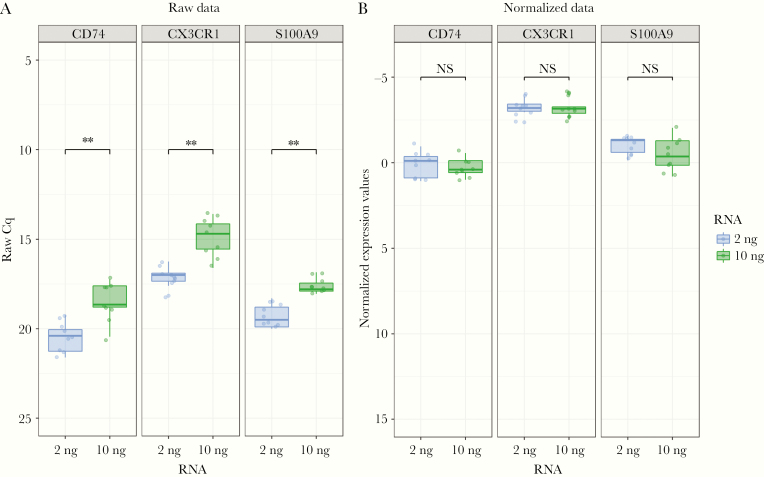
Evaluation of data normalization. The raw quantification cycle (Cq) values and normalized expression values are expressed in an inverted y-axis scale to facilitate interpretation. Two quantities of RNA samples from 10 healthy volunteers were extracted from PAXgene tubes and directly injected in the immune profiling panel pouch and tested in FilmArray. The results were tested for significance using paired Wilcoxon signed-rank test. *A*, Raw Cq values of 10 healthy volunteers expressed as boxplots showing the 2 RNA quantities tested: 2 ng (blue) and 10 ng (green). *B*, Results after Cq normalization with no significant difference observed between 2 quantities in the respective marker. **P < .05; NS, not significant.

The intended use of the IPP tool is to semiquantify immune response markers from whole blood. The use of whole blood samples might have different cell counts among patients leading to different RNA inputs, which might alter the signal. Normalization of the assays using internal reference genes could correct the signal to account for the different cell counts and would still highlight the markers’ modulation in different patient groups. Ten healthy volunteers were analyzed to assess the effectiveness of the normalization strategy. Two RNA inputs (2 ng and 10 ng) were tested and the expression signals were represented in boxplots before and after normalization. Figure 5 illustrates 3 target genes (CD74, CX3CR1, and S100A9 selected as representative of the data). Figure 5A shows a significant difference in expression level (*P* < .01) when analyzing the raw Cq values, which refer to the RNA quantities used as input (as previously inspected in the linearity study). Figure 5B shows the same data after normalization with internal reference genes. The use of normalization corrected the different RNA inputs and medians were not different.

### Clinical Sample Assessment With IPP

**Figure 6.**
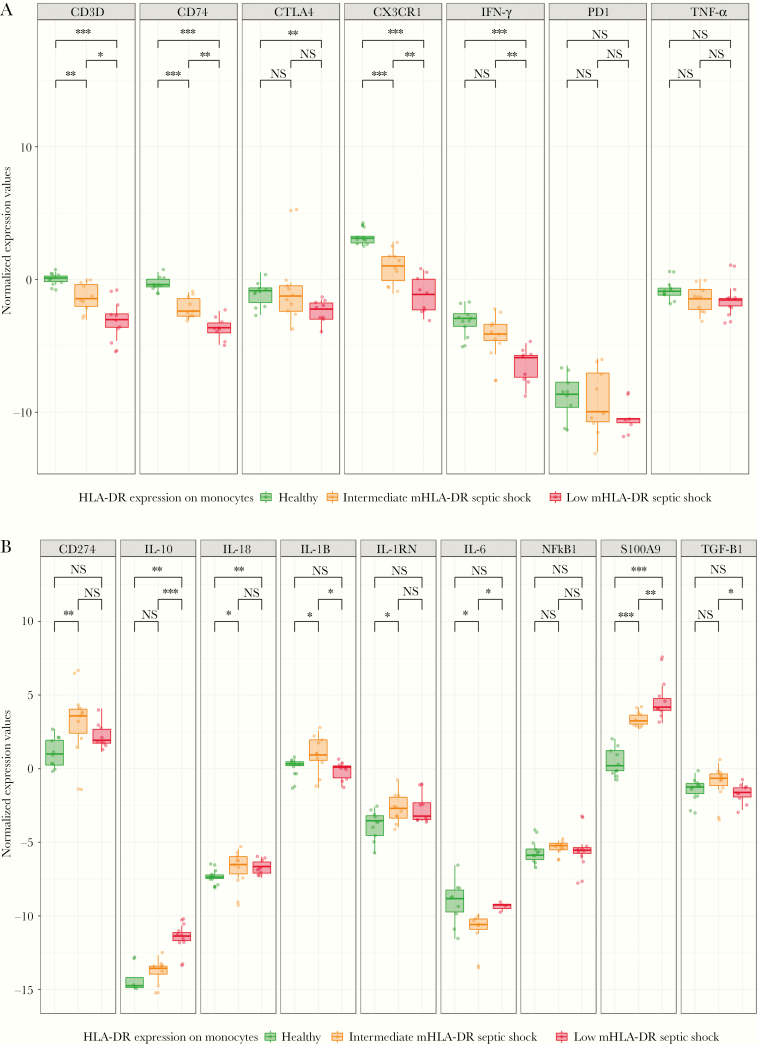
Testing the immune profiling panel on 10 healthy volunteers and 20 patients with septic shock. The y-axes representing normalized values are inverted to facilitate interpretation. A quantity of 10 ng of each RNA sample was injected directly in the immune profiling panel pouches. Normalized expression values were compared across groups using Mann–Whitney *U* test for significance. Green, healthy volunteers; orange, intermediate monocyte human leukocyte antigen-DR (mHLA-DR) in septic shock patients; red, low mHLA-DR in septic shock patients. *A*, Markers that were down-modulated in the patient groups compared to the healthy volunteers. *B*, Markers that were up-modulated in patients versus healthy volunteers. *P < .05; **P < .01; ***P < .001; NS: *P* > .05.

To ensure that after normalization, the expected variable expression of markers between patients with different immune status and healthy volunteers were conserved, we ran a proof-of-concept analysis on 10 healthy volunteers against 20 patients with septic shock stratified using mHLA-DR. Samples tested with IPP showed differential expressions of the target genes across the 3 tested populations. Figure 6A shows 6 genes that were down-modulated in patients compared to healthy volunteers. A significant difference between patients and healthy volunteers was observed in CD74, CX3CR1, CD3D, CTLA-4, and IFN-γ but not TNF. All of the previous modulations are hallmarks of sepsis and can be observed in both septic shock groups, intermediate and low mHLA-DR expression, with more altered expression in the latter. Figure 6B shows 4 assays (IL-18, IL-10, IL-1RN, and S100A9) that were significantly up-modulated in patients. Interestingly, the use of the stratified samples showed the ability of the IPP tool to clearly distinguish between healthy volunteers and patients with various degrees of immune alterations that specifically identified the immunosuppressed profiles.

## DISCUSSION

The IPP is a new molecular multiplex tool that uses the FilmArray system to provide a transcriptomic immune profile of critically ill patients in the ICU. The analytical assessment proved the ability of the selected panel to measure immune-related gene expression from whole blood in both healthy patients and those with septic shock.

The FilmArray microbiology syndromic panels have been reported in literature for their robust and reproducible results to provide qualitative detection of pathogens [27, 28, 34]. In the IPP tool, an alternative approach was sought, and we demonstrated the ability of the platform to semiquantify immune-related host-response markers. Even though the capacity of the pouch reaches up to 45 assays, we chose to use only 16 target genes and test 8 reference genes (Figure 1) as a prototype.

The IPP pouches were analytically evaluated for repeatability and the assay’s linearity and compared to qPCR, which is a well-established gold standard for analyzing the transcriptome from blood. As per the Minimum Information for Publication of Quantitative Real-Time PCR Experiments (MIQE) guidelines and recommendations, the intra-assay variability in qPCR could be assessed using SD or variance in Cq values [35]. To this end, we assessed the intra-assay variability of the IPP tool in 4 different batches. Most of the assays had a low variance, except for SDHA, which was highly variable (Figure 2) and recorded the highest coefficient of variation (19%, data not shown). All assays were further investigated in the linearity study (Figure 3) in a 3-log linear range of RNA inputs. We confirmed that the majority of the markers in the panel had an *R*^2^ ranging from 0.8 to 0.95. These values are slightly lower than the recommended *R*^2^ (≥ 0.98) for classic qPCR [36]. However, the qPCR recommendations only address a 1-step amplification PCR, whereas the *R*^2^ presented here cover the whole process from sample input to result. Equivalence studies to classic qPCR showed that the assay results were concordant with assays from IPP with a low bias for CX3CR1 (Figure 4), which was corrected by normalization. The observed *R*^2^ and bias might be explained by several reasons such as the integration of several steps in the platform that include reverse transcription and multiplex amplification, followed by a dilution step just before the second round of nested PCR. The integration of all these steps in 1 consumable pouch might slightly influence the quantity of RNA transferred from 1 step to the next and could affect the signal at the end of the run. Other factors that contribute to the observed bias might include the multiplexing environment of the platform, where some primer–primer interaction might occur in the first multiplex PCR step. The *R*^2^ might also be affected by the natural diversity of expression profiles among the healthy volunteer samples obtained from the blood bank. All of these factors make the standard guidelines more adaptable to classic qPCR and are partially applicable to the FilmArray System assessment. Nevertheless, the achieved linearity is acceptable and allows assays’ semiquantification of mRNA from whole blood. Based on the analyses, the assays SDHA, GAPDH, and ACTB were discarded and were not included in the later assessment steps as they likely have a design or compatibility issues. The rest of the assays that were identified in the variability study or had an *R*^2^ lower than 0.8 will be either redesigned or removed from the next version of the tool. Since the intended test specimen is whole blood, the effectiveness of signal normalization was confirmed by the successful correction of the varying RNA input among individuals that can influence the RNA quantity within the sample (Figure 5).

The choice of testing sepsis shock samples at days 3–4 was based on previous studies, which showed most of the sepsis-related deaths (70%) occurred after the first 3 days of sepsis onset due to persistent immunosuppression [37, 38]. A decrease in mHLA-DR expression measured by flow cytometry is widely accepted as a marker of immunosuppression in critically ill patients [3, 29], where an mHLA-DR expression < 30% was often associated mortality and secondary infections [29, 39]. A decrease in mHLA-DR expression was reported between days 1–2 and days 3–4 and was associated with acquiring secondary infections [38]. Peronnet et al also reported low expression levels of CD74 and IL-10 mRNA in whole blood of septic shock patients at day 3, which were also linked to ICU-acquired infections [40]. When IPP was tested on healthy and septic shock patient samples on days 3–4 (Figure 6), a precise stratification was possible between patients and healthy volunteers based on the differential expression of the panel. For instance, the panel successfully discriminated low mHLA-DR patients who suffer from a profound immune dysfunction in the intermediate mHLA-DR patients. This was highlighted by the down-modulation of CD74 and CX3CR1, CD3D, and CTLA-4 in patients, markers that are affiliated to the innate and adaptive immune responses, respectively. Other immune dysfunctions that were observed included alterations in both pro- and anti-inflammatory markers (IL-18, IL-10, and IFN- γ). These markers were reported in literature as hallmarks of sepsis syndrome and are indicators of an immunosuppressed profile in septic shock patients [2]. The importance of having an immune profiling panel lies in the valuable information provided by the tool about several aspects of the immune response that cannot be identified by measuring only 1 cytokine or a unique flow cytometry marker such as HLA-DR.

Recent studies attempted to potentially stratify sepsis in a patient population based on a microfluidic biochip that quantifies CD64 from circulating neutrophils in the blood and enumerates lymphocytes using only 10 µL of blood from patients in 30 minutes [41]. This approach is rather appealing, but due to the heterogeneity of immune responses in sepsis patients, both the diversity and number of addressed biomarkers may prove to be key and allow precise stratification. Similarly, interesting work by Morris et al based on a 4-hour flow cytometry protocol assessed CD88, percentage of regulatory T cells, and mHLA-DR expression. They demonstrated the potential prediction of secondary infections in septic patients [6]. However, the main challenge in using flow cytometry is the need for skilled personnel to operate and interpret the results and is often unavailable during night shifts. Flow cytometry is hard to standardize across centers and requires the presence of well-equipped laboratories, which is not the case in most hospitals. These research efforts reinforce the need for a tool such as IPP in the ICU, as it includes a panel of markers that cover diverse immune functions and could identify different patient profiles. The fact that FilmArray is a fully automated and closed system, with only 2 minutes of hands-on time, limits the risk of variability and facilitates its implementation. The use of whole blood as an input and the availability of results within the hour makes it possible to be installed at a central laboratory, thus making it accessible 24/7.

In this pilot study, we had several limitations such as the small sample size of patient groups, and most of our technical evaluations were on healthy volunteers. In addition, markers’ performance such as validity and ability to predict clinical outcomes still needs to be addressed in a dedicated clinical cohort. The further addition of assays in the next pouch versions will require a full analytical validation and evaluating the compatibility of all primers in the multiplexing environment. There is still a gap in the assessment of the immune status of ICU patients, and physicians have no available tools that can capture the heterogeneity of sepsis patients and allow patient stratification. Monitoring key aspects of the immune response and identifying critically ill patients with altered immune status will be instrumental in implementing new immunotherapies to improve the overall patient management [42]. Our upcoming goal is to increase the multiplexing capacity of the panel and achieve a highly informative tool reflecting the immune status of a patient at a given time. The final IPP panel will have a simplified readout that may one day be integrated into routine clinical practice. This will provide personalized information for each patient and will enable clinicians to precisely manage critically ill patients according to their immune profile.

## Supplementary Data

Supplementary materials are available at *The Journal of Infectious Diseases* online. Consisting of data provided by the authors to benefit the reader, the posted materials are not copyedited and are the sole responsibility of the authors, so questions or comments should be addressed to the corresponding author.

jiaa248_suppl_Supplemental_Figure-1

jiaa248_suppl_Supplemental_Table-1

## Notes

***Author contributions.*** All authors contributed to the manuscript structuring and critical revision for important intellectual content, and read and approved the final manuscript. L. V., A. B., F. M., J. Y. M., and J. T. designed the experiments. D. M. T. and A. B. performed the experiments and the statistical analyses. D. M. T. drafted the manuscript. D. M. T., L. V., and J. T. made substantial contributions to the conception and design of the study and final data interpretation. All authors approved the final manuscript.

***Acknowledgments.*** We acknowledge Estelle Peronnet, Elisabeth Cerrato, and Boris Meunier for their technical support and sharing their expertise to enrich our study. We extend our gratitude to the ImmunoSepsis-1 study group for providing us with the septic shock clinical samples.

***Financial support.*** This work was supported by bioMérieux and Hospices Civils de Lyon. D. M. T., L. V., A. P., F. M., and J. T. are part of the European Sepsis Academy funded by the European Union’s Horizon-2020 research and innovation program under grant agreement number 676129 from the Marie Skłodowska-Curie Innovative Training Networks.

***Supplement sponsorship.*** This supplement is sponsored by bioMérieux, the Gordon and Betty Moore Foundation and Beckman Coulter.

***Potential conflicts of interest.*** D. M. T., L. V., F. V., T. R., G. M., S. B., A. P., V. M., K. B.-P., F. M., and J. T. work in a joint research unit, co-funded by the Hospices Civils de Lyon and bioMérieux. A. P. and G. M. are co-inventors in patent applications on markers included into the test. bioFire (a bioMérieux company) holds patents on the technology; this does not alter the authors’ adherence to all the policies on sharing data and materials. All other authors report no potential conflicts of interest.
