# Supplementary material for: Immune Profiling Panel: A Proof-of-Concept Study of a New Multiplex Molecular Tool to Assess the Immune Status of Critically Ill Patients
Source: J Infect Dis. 2020 Jul 21;222(Suppl 2):S84–95. doi: 10.1093/infdis/jiaa248 (PMC7372218; doi:10.1093/infdis/jiaa248)
Supplement: jiaa248_suppl_Supplemental_Figure-1 [file jiaa248_suppl_supplemental_figure-1.docx]

**Supplementary Figure 1 The FilmArray pouch**

**
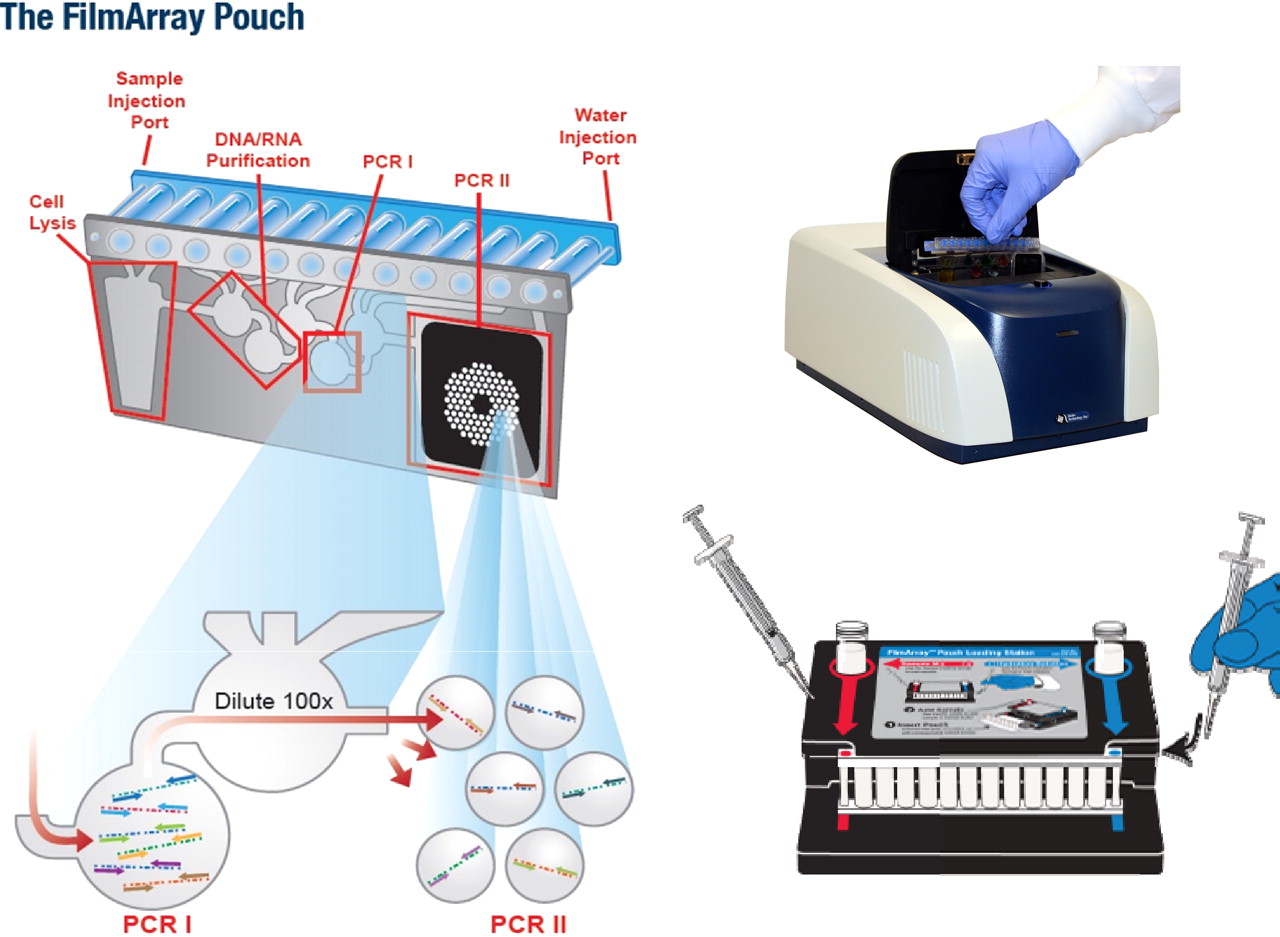
**

The FilmArray is a fully integrated system that combines an automated sample preparation, nucleic acid extraction followed by PCR-based detection of multiple targets from a single unprocessed sample in one hour. The FilmArray kit is supplied with a consumable enclosed pouch containing all the lyophilized biochemical reagents that are sealed under vacuum.

The reagents in the pouch are hydrated by injecting the hydration solution supplied with the kit into the hydration port, meanwhile, 100µl of the blood sample is mixed with two volumes of the denaturing sample buffer and injected into the pouch through the sample injection port. When the pouch is launched in the FilmArray instrument, the nucleic acids are extracted and purified from blood cells then transferred to stage one PCR1. The first stage PCR1 is the multiplexing step, where the nucleic acids are amplified using numerous primer pairs followed by a dilution step then mixed with fresh PCR reagents to be finally transferred to stage two PCR2. The second stage PCR2 is a nested quantitative PCR where amplicons are specifically amplified into 96-array nested singleplex reaction containing one primer per well. Simultaneously, the quantification cycle value (Cq) for each target is detected by the changes in fluorescence using double-stranded DNA binding dye LC green plus (Idaho Technology, USA).
