# Supplementary material for: Immune Profiling Panel: A Proof-of-Concept Study of a New Multiplex Molecular Tool to Assess the Immune Status of Critically Ill Patients
Source: J Infect Dis. 2020 Jul 21;222(Suppl 2):S84–95. doi: 10.1093/infdis/jiaa248 (PMC7372218; doi:10.1093/infdis/jiaa248)
Supplement: jiaa248_suppl_Supplemental_Table-1 [file jiaa248_suppl_supplemental_table-1.docx]

**Supplementary Table 1. Linearity of mRNA marker quantification was associated through R^2^ coefficients from linear regression over RNA quantity used in input.**

| 1. Target Marker | R^2^ |  | 1. Reference Marker | R^2^ |
| --- | --- | --- | --- | --- |
| IL6 | 0.51 |  | ACTB | 0.63 |
| PD1 | 0.63 |  | SDHA | 0.74 |
| IL10 | 0.80 |  | GAPDH | 0.78 |
| IL1RN | 0.82 |  | DECR1 | 0.86 |
| TGFB1 | 0.85 |  | TRAP1 | 0.89 |
| CD74 | 0.85 |  | PPIB | 0.92 |
| IFNg | 0.86 |  | HPRT1 | 0.93 |
| IL1B | 0.87 |  | FPGS | 0.94 |
| CD3D | 0.87 |  |  |  |
| IL18 | 0.88 |  |  |  |
| NFkB | 0.88 |  |  |  |
| CTLA4 | 0.89 |  |  |  |
| S100A9 | 0.91 |  |  |  |
| CD274 | 0.91 |  |  |  |
| CX3CR1 | 0.92 |  |  |  |
| TNFa | 0.93 |  |  |  |
